# Letter upon Yellow Fever Inoculation

**Published:** 1894-03

**Authors:** W. H. Doughty

**Affiliations:** Augusta, Ga.


### LETTER UPON YELLOW FEVER INOCULATION.

AUGUSTA, GA., February 16, 1894.

*Dr. J. McFadden Gaston, Atlanta, Ga.:*

DEAR DOCTOR—I have just read your "Open Letter upon the Prevention of Yellow Fever by Inoculation," in the January number of THE SOUTHERN MEDICAL RECORD, reprinted from the *Journal of the American Medical Association* of January 5th.

If by this writing I can "hold up Moses' hand," I shall feel repaid, however unnecessary that aid may appear to one who, in the strength of his resources, proclaims his ability and purpose to present "before the medical profession at an early day, facts in his possession confirmatory of *all* that has been alleged in favor of the results of inoculation against yellow fever, as practiced by Domingos Friere in Brazil."

The subject is one of overwhelming importance as well as public interest. That your presentation of the data at hand will be acceptable, I need not assure you; the profession will await it with expectant interest, increased, if not prompted, by the hope that whatever prejudice to the cause of the prophylaxis of yellow fever by this means has resulted from the action of the commission may be speedily removed, and at least the hopeful attention of the scientific world be again accorded to the investigator.

I can plead an early interest in this subject, as the author of a petition signed by the Georgia Medical Society of Savannah, the Board of Health and the Academy of Medicine of this city, to the Congress of the United States in 1884, to offer a reward, "open to the world, for the discovery of the true cause or germ of yellow fever, or any certain means of affecting its prevention, destruction or harmless modification." This was done with a view to "stimulate inquiry in this direction," which seemed in the then state of knowledge the only one of promise as to the control of this epidemic disease. Dr. Friere's appearance upon the stage of action about that time was to me, as it doubtless was to others, especially interesting and timely.

Dr. Friere will be fortunate if he encounters opposition in only *official* form, for the path of independent research and discovery in such matters is a thorny one indeed, and he who pursues it needs alike the patience of Job and the "courage of his convictions." Witness Jenner, the great exemplar, of whom it is said: "During six years no members of the profession ever received more anathemas or scurrilous abuse. He was attacked by the leading physicians and surgeons of Great Britain, and persecution and ridicule so followed him that placards with caricatures of Jenner were posted throughout the streets of London and the principal towns of Great Britain. Jenner kept steadily at work and repeated his experiments until he became fully convinced that by vaccination perfect protection could be obtained against small-pox."

Science has never yet discharged the debt of obligation it justly owes to tradition and *empirical popular* observation by the discovery of vaccination through that medium. In 1771 a Holstein *schoolmaster* vaccinated three pupils, and in 1774 an English *farmer* vaccinated his wife because of his belief in the power of bovine virus as seen in his dairy-maids." It was twenty-five years later (1796) that Jenner made his first vaccination on man. This obligation can never be discharged, as the occasion that created it led to the first experimental demonstration of the now more fully established theory of the "attenuation of viruses," the vista through which hope sees in the distant future the revelation of a power over contagious epidemic diseases unauthorized by all other human means.

You foreshadow objections to Dr. Sternberg's "mode of conducting the investigation" that led to his unfavorable report. Now, without meaning to criticise this distinguished officer, it can be said that commissions of inquiry into matters of this kind, especially when prefaced by a certain degree of incredulity on the part of the individuals composing them as to the observer whose work is to be examined, or incredibility as to the subject under investigation, cannot, or ought not to, be taken as final. As evidence of this I need only refer to that admirable "Report on the Cholera in Europe and India," made in 1890 by Dr. E. O. Shakspeare, A. M., M. D., Ph. D., of Philadelphia, who was appointed by the United States Government Special Commissioner for that purpose. That report, commendable alike for its impartiality, ability and fidelity to the trust imposed by the government which he represented, is a model of its kind.

Among other matters of investigation incident to this mission was that of the anti-choleraic inoculations practiced by Dr. Ferran, of Spain. Whether of value or not, they had attracted the attention of Europe and the scientific world. Commissions were sent by different countries to look into the merits of this so-called discovery. Among them one from France, headed by Brouardel. Appointed by the Minister of Commerce, his commission also bore a letter to Dr. Ferran from the immortal Pasteur, before whom both science and commerce in their approaches may be fitly presented in a kneeling, if not worshipping, attitude, with upturned palms ready to receive the gifts of God transmitted through this medium.

Reading the report of this commission one is lamentably impressed with the shortcomings of human nature. Because Dr. Ferran saw proper to withhold "his mode of attenuation of the cholera virus," although offering every facility for the examination of the "vaccinal liquid" in his laboratory, the commission made a report disparaging to his preparation and outfit for the work; intimated a want of "honesty of the man," and while admitting that the "inoculations upon man appear harmless," yet represented him as the subject of an imprudent "infatuation" which committed him to its premature trial on man, in extenuation of their position pleading the example of Jenner, who "hesitated nine years before daring to inoculate James Phipps, May 14th, 1796." If this commission had simply reported their inability to pursue the objects of their inquiry on account of the refusal of Dr. Ferran to disclose or subject to examination his mode of preparation of virus, its duty would have been discharged; but in reflecting upon Dr. Ferran in the above manner, it sacrificed its own prestige and influence to bitter prejudice or disappointment.

The United States Commissioner says: "After having read so much in the current literature of the day about the ignorance of Dr. Ferran of the modern methods of research among bacteria, and of his inability to make decent microscopic preparations, and of his absolute ignorance of the method of staining, I confess that it was with some surprise that I witnessed the facility with which he performed all these operations—a facility which indicated the habitual practice of no mean skill in the performance of these somewhat delicate operations; and my astonishment when for the first time I examined with the Hartnack microscope one of the cover-glass preparations which I had seen him make. Honesty and regard for fair dealing require me to say that if there are more beautifully stained microscopic preparations of bacteria, especially of the comma-bacillus of Koch, and of the Finkler and Prior bacillus, in Europe, I have never seen them." "I found him to be a quiet, reserved, courteous, intelligent, and generally well-informed physician. The impression which I formed of his theoretical knowledge of bacteria and of the modern methods of research in the field of natural history, have compared very favorably with those of most of the leading bacteriologists with whom I have come in contact." (Page 714.)

Moreover, as you propose to furnish data confirmatory of the discredited results of Dr. Friere's yellow fever inoculations, it may interest you if I quote in this connection Dr. Shakspeare's statement of the results of the anti-choleraic inoculations in Spain. After citing statistics he adds: "It appears therefore that among the population of villages wherein anti-choleraic inoculations had been more or less extensively performed, the liability of the inoculated to attacks of cholera was 6.06 times less than that of the non-inoculated, whilst the liability of the inoculated to death by cholera was 9.87 times less than that of the non-inoculated." (Page 715.)

Between Drs. Ferran and Friere there exists a close analogy in their respective spheres of observation. Hitherto unknown members of the profession, and alike bold in the practical application of the results of their researches, they first fell heir to incredulity, if not prejudice, in the minds of the profession, and invited a burden of proof positive as to the value of their observations, which, to say the least, it is extremely difficult to furnish under present circumstances. Their tasks embraced not merely the finding of a missing link in the development of the bacillus anthracis, or the isolation of the germ of cholera or tuberculosis, like Koch, but much more, the immediate practical application and use of a virus for the prevention and modification of the two remaining unmitigated scourges of mankind—yellow fever and cholera. The discovery of the bacillus-tuberculosis and the comma-bacillus has done little or nothing for the treatment of the diseases of which they are the respective causes, while inoculations against cholera and yellow fever become from their very nature and objects, the full equivalent of the highest therapeutical results. A stronger proof, more difficult to furnish than the demonstration of a micro-organism, is necessary with the latter, and even if brought to naught in the end, may not be without useful lessons to future investigators. It will doubtless prove an acceptible duty to you to show the full value and usefulness of Dr. Friere's labors and researches.

Yours truly,

W. H. DOUGHTY.

